# Temporal Relationship Between Changes in Serum Calcium and Hypercholesteremia and Its Impact on Future Brachial-Ankle Pulse Wave Velocity Levels

**DOI:** 10.3389/fnut.2021.754358

**Published:** 2021-11-17

**Authors:** Xing Meng, Tianshu Han, Wenbo Jiang, Fengli Dong, Hongxue Sun, Wei Wei, Yageng Yan

**Affiliations:** ^1^Division of Clinical Nutrition, The First Affiliated Hospital of Harbin Medical University, Harbin, China; ^2^National Key Discipline, Department of Nutrition and Food Hygiene, School of Public Health, Harbin Medical University, Harbin, China; ^3^Department of Neurology, The First Affiliated Hospital of Harbin Medical University, Harbin, China

**Keywords:** serum calcium, cholesterol, cardiovascular diseases, brachial-ankle pulse wave velocity, cohort study

## Abstract

**Background:** The high levels of serum calcium and cholesterol are the important risk factors of cardiovascular disease (CVD), which frequently influence each other during the development of CVD. However, few studies have examined their temporal relationship to confirm the precursor, and it is still largely unknown whether and how their temporal relationship would influence the development of CVD. This study aimed to establish the temporal relationship between the changes in serum calcium and cholesterol using the longitudinal cohort data, and examine whether this temporal relationship influenced the arterial elasticity indicated by brachial-ankle pulse wave velocity (baPWV).

**Methods:** This is a cohort study with a sample of 3,292 Chinese participants (aged 20–74 years) with 5.7 years follow-up. Serum calcium and cholesterol were measured at baseline and follow-up survey. The cross-lagged path analysis was used to examine their temporal relationship, and mediation analysis was performed to evaluate the potential mediating effect.

**Results:** The cross-lagged path coefficients (β_2_ values) from baseline serum calcium to follow-up cholesterol was significantly greater than the path coefficients (β_1_ values) from baseline cholesterol to follow-up serum calcium (β_2_ = 0.110 vs. β_1_ = 0.047; *P* = 0.010) after adjusting for the multiple covariates. The path coefficients from baseline serum calcium to follow-up cholesterol in the participants with high baPWV was significantly greater than the participants with low baPWV (β_2_ = 0.155 for high baPWV and β_2_ = 0.077 for low baPWV, *P* = 0.028 for the difference between the β_2_ values). Moreover, cholesterol partially mediated the association between the higher serum calcium and greater subsequent baPWV values, the percentage of the total effect mediated by cholesterol was estimated at 21.7%.

**Conclusion:** Our findings indicate that increased serum calcium precedes increased in serum cholesterol, and this temporal relationship may contribute to the development of higher baPWV levels.

## Introduction

Hypercalcemia and hypercholesterolemia are the two important risk factors for arterial stiffness or cardiovascular disease (CVD) (1–3). A few cross-sectional studies have documented the significant association between the higher levels of serum calcium and cholesterol, indicating that the high levels of serum calcium and cholesterol are also closely related with each other (4, 5). Further, one animal study demonstrated that increased in serum calcium could elevate the circulating cholesterol through reducing its catabolism *via* G-protein coupled estrogen receptor and transient receptor potential canonical 1-dependent pathway (6), whereas the other animal study found that cholesterol could affect the serum calcium levels by decreased bone calcium, calcium redistribution in bone, and change of bone metabolism (7), indicating that the serum calcium and cholesterol also could physiologically influence each other. These evidence collectively suggested that the dynamic of the association between the changes in serum calcium and cholesterol is probably far from straightforward because the changes in either one may precede changes in the other. Therefore, it is important to establish the temporal relationship between the serum calcium and cholesterol to confirm which one is the precursor or the temporal relation is bidirectional because it may result in the different intervention strategies for reducing the burden of CVD.

The cross-lagged path analysis is a statistical method that is frequently used to dissect the temporal relationship among the closely related variables. A series of recent studies have successful used this method to establish the temporal relationship between the two closely related biochemical indicators for confirming the priority therapeutic targets for the prevention of diabetes, hypertension, or CVD (8–10). Therefore, this study aimed to establish the temporal relationship between the changes in serum calcium and cholesterol in a Chinese longitudinal cohort data. Moreover, once we could confirm the precursor in this temporal relationship, we would examine whether and how this temporal relationship involved in the increased in the brachial-ankle pulse wave velocity (baPWV) using the mediation analysis because the baPWV is directly related to arterial stiffness, which is considered to be the “gold standard” method for the measurement of arterial stiffness, and is currently well recognized as an ideal indicator for assessing early CVDs (11–13).

## Methods

### Study Population

Data were drawn from the Harbin Cohort Study on Diet, Nutrition, and Chronic Non-communicable Disease Study (HDNNCDS) (Trial Registration: ChiCTR-ECH-12002721), carried out in 2010 with a follow-up in 2015–2016 (14). The HDNNCDS covered seven urban administrative regions of Harbin. Each region was divided into three strata according to their financial situation, and a total of 42 communities were randomly selected from each stratum in each administrative region by employing a stratified multistage random cluster sampling design. A total of 9,734 subjects participated in the HDNNCDS in the 2010 baseline survey. During 2015–2016, 8,913 subjects completed the first in-person follow-up survey with a response rate of 91.6%. In this study, we included 3,292 participants who measured the levels of serum calcium and cholesterol at the baseline and follow-up, as well as baPWV during the follow-up survey without the CVD or any missing information in the questionnaire at the baseline, were included in this analysis, with an average follow-up period of 5.7 years. The HDNNCDS were approved reviewed by the institutional review boards of Harbin Medical University, and were conducted in accordance with the Declaration of Helsinki. The written consents were obtained from all the participants.

### Questionnaire Survey

Data on the dietary habits and lifestyles, physical activity level, smoking, and drinking status were collected using the face-to-face questionnaires answered by the participants. Regular exercise was defined as any type of recreational or sports other than walking performed 3 or more days per week for at least 30 min. The current smokers were defined as those who smoked at least 100 cigarettes in a lifetime or smoked every day. The current drinkers were defined as those who consumed ≥1 alcoholic drink each month in the past 1 year before the survey.

### Anthropometric Measurements and Biochemical Analyses

The height and weight were measured with the participants wearing light, thin clothing, and no shoes. The height and weight were measured to the nearest 0.1 cm and 0.1 kg, respectively. Body mass index (BMI) was calculated as weight (kg) divided by the square of the height in meters (m^2^). The blood samples were collected from all the participants at both the baseline and follow-up surveys. The blood samples were collected and immediately centrifuged at 2,500 g for 15 min to obtain serum and were then cooled. After collection, the plasma samples were kept in a portable, insulated bag with ice packs (at about 0–4°C) and were processed within 6 h for long-term storage at −80°C. The blood sample repositories for the study are equipped with the appropriate alarm systems and emergency electricity backup to prevent the accidental thawing. Serum calcium, total cholesterol, triglyceride (TG), low-density lipoprotein cholesterol (LDL-C), and high-density lipoprotein cholesterol (HDL-C) were measured using an automatic biochemistry analyzer (Hitachi 7100, Tokyo, Japan). The chemical reagents of analytical grade were used (Wako Pure Chemical Industries Ltd, Tokyo, Japan). All the analytical methods were controlled according to the instructions from the manufacturer by preventive maintenance, function checks, calibration, and quality control.

### Measurement of BaPWV

The baPWV measurements were made using an automated system (BP-203RPE; Omron Colin, Tokyo, Japan). The technicians from our center were all similarly trained and accredited. The subjects were instructed not to take any medications, alcohol, or caffeine on the day of the examination. After rest in supine position for 10 min, the subjects remained in supine position while the cuff is attached to the plethysmographic and oscillometric sensors placed around both the arms and ankles. The baPWV was measured two times automatically and the mean of the right and left PWVs was calculated as the representative baPWV (15).

### Statistical Analysis

The serum calcium and cholesterol were measured at two time points, which is a typically cross-lagged panel design. To improve the normality of the distribution serum calcium and cholesterol were log transformed at the baseline and follow-up. The generalized linear models were performed to test differences in the continuous variables between the high baPWV group and low baPWV group.

The baseline serum calcium and cholesterol were grouped by quintiles, and the differences between the follow-up serum calcium or cholesterol and the baseline serum calcium or cholesterol were calculated (follow-up serum calcium or cholesterol—baseline serum calcium or cholesterol), which were then grouped by the median values. The logistic regression models were performed to calculate the relative ratio (RR) and 95% *CI* with adjustment for age, sex, BMI, smoking, alcohol consumption, regular exercise, marriage, caloric intake, family history of cardiovascular disease, TG, HDL-C, LDL-C, and drug use for hypertension or dyslipidemia.

The serum calcium and cholesterol at two time points were adjusted for age, sex, BMI, smoking, alcohol consumption, regular exercise, marriage, caloric intake, family history of cardiovascular disease, TG, HDL-C, LDL-C, and drug use for hypertension or dyslipidemia using a regression residual analysis before the cross-lagged path analysis, and further standardized by the *Z* transformation (mean = 0; SD = 1). The theoretical model of cross-lagged path analysis assumes that if the relationship between the changes in serum calcium and cholesterol was bidirectional, then they can predict each other, and their cross-lagged path coefficients will not be significantly different. However, if the changes in serum calcium and cholesterol have an underlying causal relationship, then the causal variable can predict the consequent variable, and the cross-lagged path coefficient of the causal variable will be significantly greater than that of the consequent variable. The path coefficients for β_1_ represented the baseline cholesterol on subsequent serum calcium, and the path coefficients for β_2_ represented the baseline serum calcium on subsequent cholesterol. Fisher's Z test was used to test the difference between β_1_ and β_2_ derived from the standardized variables. The root mean square residual (RMR) for which values < 0.05 and comparative fitness index (CFI) for which values >0.90 reflect a good fit (16, 17). The receiver-operator characteristic (ROC) curves were used to analyze the predictive accuracy of baPWV. Afterward, the baPWV with the highest sum of sensitivity and specificity was identified as the cut-off point.

After determining the temporal relationship between serum calcium and cholesterol, a causal mediation model was constructed to examine whether the association of serum calcium with value of baPWV was mediated by cholesterol after adjusting for age, sex, BMI, smoking, alcohol consumption, regular exercise, marriage, caloric intake, family history of CVD, TG, HDL-C, LDL-C, and drug use for hypertension or dyslipidemia. The baseline serum calcium was the predictor variable (X); follow-up cholesterol was mediator (M); follow-up value of baPWV was the outcome variable (Y). A mediation analysis was performed using the R package Lavaan (18). The analyses were performed by using R 2.15.3 (http://www.r-project.org/) and LISREL 8.52. A two-sided *p*-value < 0.05 was considered statistically significant.

## Results

### The Characteristics of Study Participants

This analysis consists of 3,292 participants who measured serum calcium and cholesterol at the baseline and follow-up. The characteristics of the studied population by the high baPWV group and low baPWV group are presented in Table 1. Compared with the participants in the group of low baPWV, the participants in the high baPWV group are older (46.2 vs. 53.4 years, *P* < 0.001), and have greater BMI (24.6 vs. 25.3 kg/m^2^, *P* < 0.001), energy intake (2,360 vs. 2,394 kcal/d, *P* = 0.009), the serum calcium levels (2.26 vs. 2.28 mmol/L, *P* < 0.001),cholesterol level (4.70 vs. 4.96 mmol/L, *P* < 0.001), TG (1.62 vs. 2.01 mmol/L, *P* < 0.001), LDL-C (2.94 vs. 3.08 mmol/L, *P* < 0.001), higher smoking rate (15.2 vs. 18.5%, *P* < 0.01), exercise frequency (44.9% vs. 49.6%, *P* < 0.01), drug use for hypertension (5.9 vs. 15.4%, *P* < 0.001), and dyslipidemia (25.5 vs. 34.6%, *P* < 0.001) rate at the baseline. The high baPWV group have greater serum calcium levels and cholesterol levels than the low baPWV group at follow-up.

**Table 1:** The characteristics regarding the study variables at the baseline and follow-up by baPWV in the Harbin Cohort Study on Diet, Nutrition, and Chronic Non-communicable Disease Study (HDNNCDS).

	**Low baPWV group *N* = 2042**	**High baPWV group*N* = 1250**	*P*-value^*^	**Total *N* = 3,292**
Baseline
Age (years)	46.2 ± 8.9	53.4 ± 8.6	<0.001	48.9 ± 9.5
Male [*n* (%)]	643 (31.5)	559 (44.7)	<0.001	1,202 (36.5)
BMI (kg/m^2^)	24.6 ± 3.5	25.3 ± 3.4	<0.001	24.8 ± 3.5
Energy (kcal/d)	2,360 ± 872	2,394 ± 915	0.009	2,373 ± 889
Smokers [*n* (%)]	311 (15.2)	231 (18.5)	<0.01	542 (16.5)
Alcohol consumption [*n* (%)]	746 (36.5)	467 (37.4)	0.63	1,213 (36.8)
Exercise habits [*n* (%)]	916 (44.9)	620 (49.6)	<0.01	1,536 (46.7)
Serum calcium (mmol/L)	2.26 ± 0.12	2.28 ± 0.11	<0.001	2.27 ± 0.12
Total cholesterol (mmol/L)	4.70 ± 1.00	4.96 ± 0.99	<0.001	4.80 ± 1.00
Triglycerides (mmol/L)	1.62 ± 1.57	2.01 ± 2.23	<0.001	1.77 ± 1.86
HDL-C (mmol/L)	1.28 ± 0.32	1.23 ± 0.31	<0.001	1.26 ± 0.32
LDL-C (mmol/L)	2.94 ± 0.83	3.08 ± 0.84	<0.001	2.99 ± 0.83
Medication for hypertension [*n* (%)]	121 (5.9)	193 (15.4)	<0.001	314 (9.5)
Medication for dyslipidemia [*n* (%)]	521 (25.5)	433 (34.6)	<0.001	954 (29.0)
Follow-up
Serum calcium (mmol/L)	2.28 ± 0.12	2.29 ± 0.14	0.012	2.29 ± 0.13
Total cholesterol (mmol/L)	4.91 ± 0.94	5.14 ± 0.94	<0.001	5.00 ± 0.95
baPWV (cm/s)	1,300.6 ± 210.1	1,877.3 ± 252.9	<0.001	1,519.6 ± 360.6

*The continuous variables are presented as the means ± SD. The generalized linear models and chi-square tests were used to probe for differences in the continuous variables (adjusted for age) and dichotomous variables*.

* *P-value for the difference of variables between the groups of brachial-ankle pulse wave velocity (baPWV) ≤ 1600.9 cm/s and baPWV > 1600.9 cm/s*.

### Temporal Relationship Between Serum Calcium and Cholesterol

**Figure 1:**
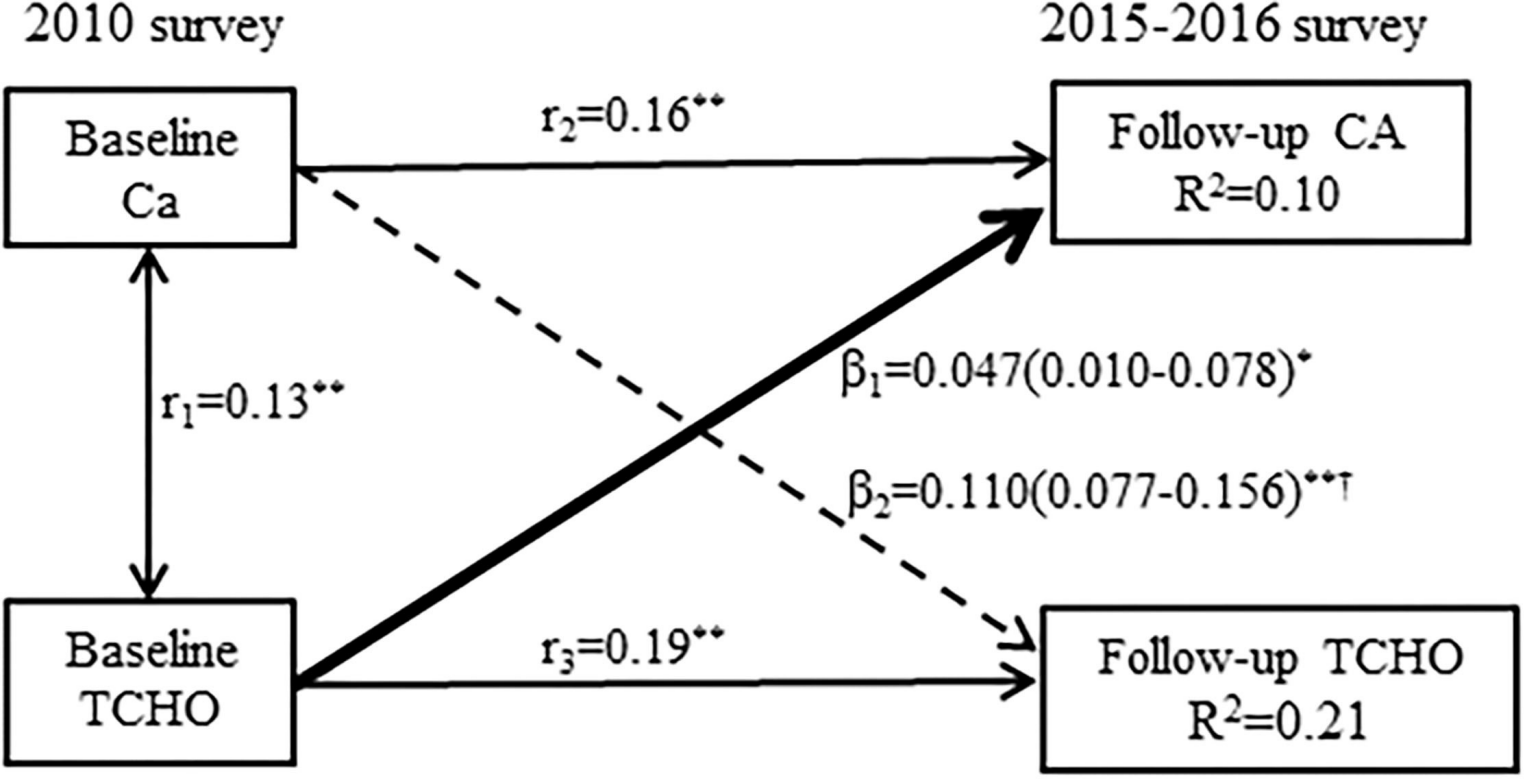
A cross-lagged path analysis of serum calcium and total cholesterol, adjusted for the covariates (covariates such as age, sex, body-mass index [BMI], smoking, alcohol consumption, regular exercise, marriage, caloric intake, family history of cardiovascular disease [CVD], triglyceride [TG], high-density lipoprotein cholesterol [HDL-C], low-density lipoprotein cholesterol [LDL-C], and drug use for hypertension or dyslipidemia) in the total sample (*N* = 3,292); β_1_ cross-lagged path coefficient from the baseline total cholesterol to follow-up serum calcium, β_2_ cross-lagged path coefficient from the baseline serum calcium to follow-up total cholesterol, *r*_1_ *r*epresents synchronous correlations, *r*_2_ and *r*_3_ represent tracking correlations, *R*^2^ variance explained. **P < 0.001 for coefficients being different from 0; ^†^difference between β_1_ and β_2_ for being different from 0.

Compared with the participants in the lowest quintile of serum calcium, the participants in the highest quintile are more likely to have greater increased in the serum cholesterol with adjustment for the potential confounders such as the baseline serum cholesterol (*RR* = 2.11, 95% *CI*: 1.74–2.55). Similarly, compared with the participants in the lowest quintile of serum cholesterol, the participants in the highest quintile are more likely to have greater increased in serum calcium (*RR* = 1.50, 95% *CI*: 1.14–1.97). These results suggested that the conventional analysis models could not dissect the temporal relationship between the changes in serum calcium and cholesterol. The cross-lagged path analysis of serum calcium and cholesterol is shown in Figure 1. The path coefficient from the baseline serum calcium to the follow-up cholesterol (β_2_ = 0.110, 95% *CI*: 0.077–0.156, *P* < 0.001) is significantly greater than the path coefficient from the baseline cholesterol to the follow-up serum calcium (β_1_ = 0.047, 95% *CI*: 0.010–0.078, *P* = 0.035) after adjusting for age, sex, BMI, smoking, alcohol consumption, regular exercise, marriage, caloric intake, family history of CVD, TG, HDL-C, LDL-C, and drug use for hypertension or dyslipidemia with *P* < 0.001 for difference between β_1_ and β_2_. The models are relatively well fitted in our analysis, RMR and CFI were 0.017 and 0.989, respectively. The variance of follow-up serum calcium and cholesterol (*R*^2^) explained by the baseline serum calcium and cholesterol are estimated at 0.10 and 0.25, respectively.

**Figure 2:**
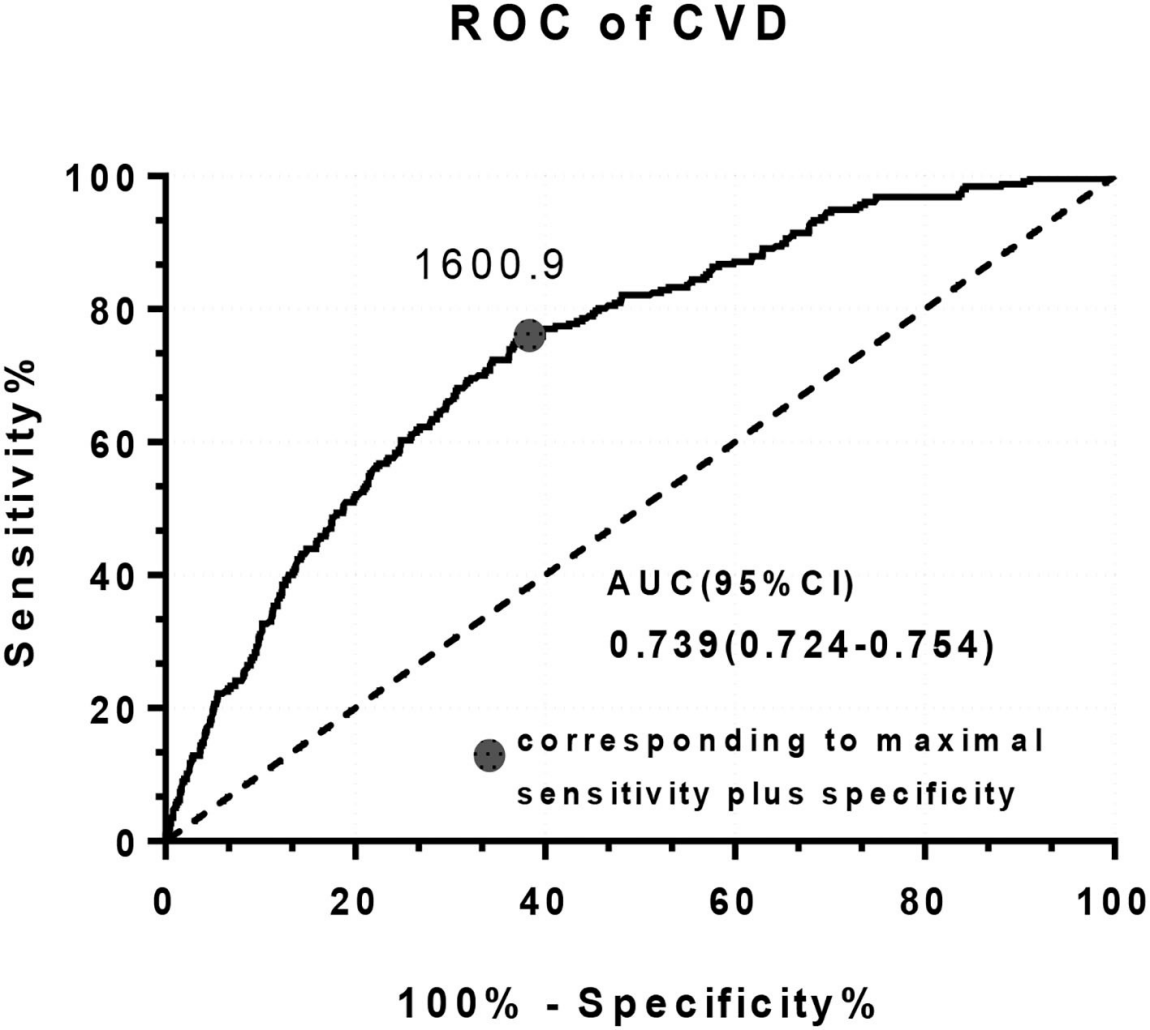
The receiver operating characteristics (ROC) curve for brachial-ankle pulse wave velocity (baPWV) to detect CVD.

Figure 2 shows the ROC curve to investigate the cut-off of the baPWV to detect the CVDs, the identification of CVD is based on the self-report and clinical diagnosis. The optimal cut-off value of baPWV is 1600.9 cm/s with sensitivity of 0.751 and specificity of 0.631, the area under the ROC curve (AUC) is 0.739 (95% *CI*: 0.724–0.754).

**Figure 3:**
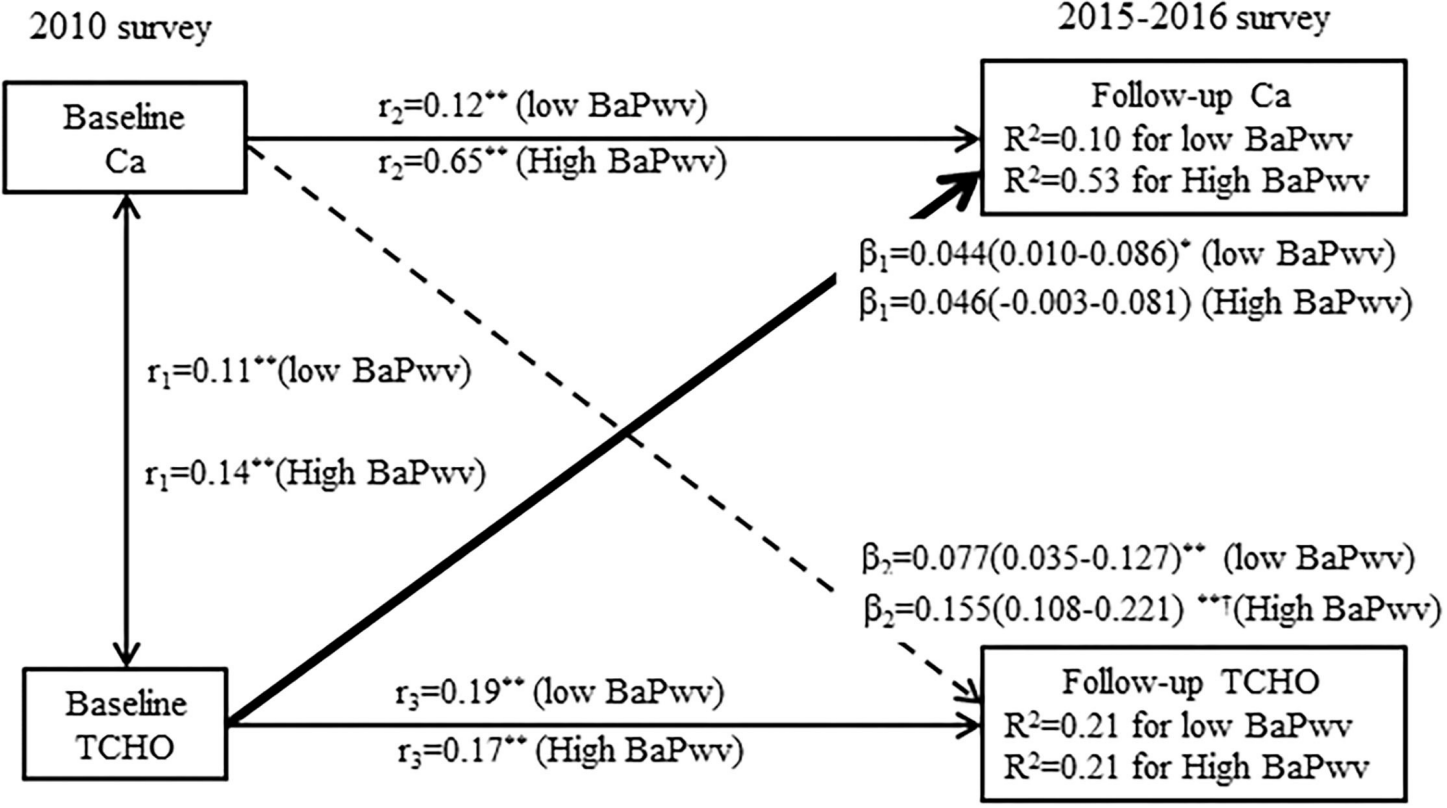
A cross-lagged path analysis of serum calcium and total cholesterol in the low baPWV group and high baPWV group, adjusted for the covariates (covariates included age, sex, BMI, smoking, alcohol consumption, regular exercise, marriage, caloric intake, family history of CVD, TG, HDL-C, LDL-C, and drug use for hypertension or dyslipidemia) in the total sample (*N* = 3,292); β_1_ and β_2_ are cross-lagged path coefficients, *r*_1_ represents the synchronous correlations, *r*_2_ and *r*_3_ represent tracking correlations, *R*^2^ variance explained. **P < 0.001 for coefficients being different from 0. ^†^difference between β_1_ and β_2_ for being different from 0.

Figure 3 presents cross-lagged path analysis models of serum calcium with cholesterol in the high baPWV group and low baPWV group after adjusting for age, sex, BMI, smoking, alcohol consumption, regular exercise, marriage, caloric intake, family history of CVD, TG, HDL-C, LDL-C, and drug use for hypertension or dyslipidemia. The unidirectional relationship from serum calcium to cholesterol is significantly greater in the participants with the high baPWV group than the participants with low baPWV group. However, the path coefficients (β_2_ = 0.155, *P* < 0.001) from the baseline serum calcium to follow-up cholesterol in the high baPWV group are greater than that (β_2_ = 0.077, *P* < 0.001) in the low baPWV group, but not significant (*P* = 0.028 for the difference between β_2_ values). The model fitting parameters are RMR = 0.013 and CFI = 0.977 in the high baPWV group, and RMR = 0.018 and CFI = 0.971 in the low baPWV group.

### Mediation Analysis

**Figure 4:**
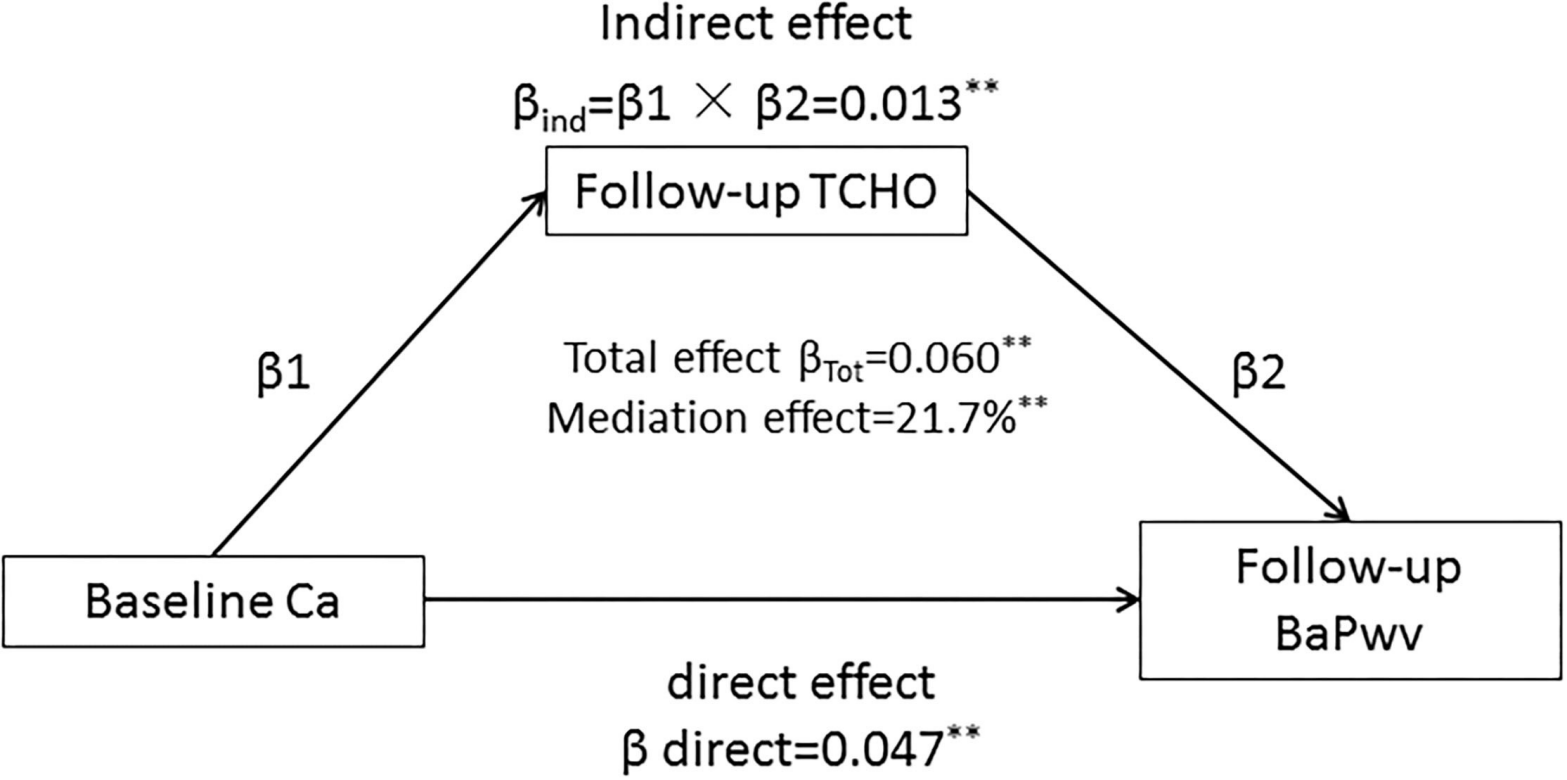
The mediation effect of follow-up cholesterol on the baseline serum calcium with future value of baPWV association with adjustment for the covariates (covariates included age, sex, BMI, smoking, alcohol consumption, regular exercise, marriage, caloric intake, family history of CVD, TG, HDL-C, LDL-C, and drug use for hypertension or dyslipidemia) in the total sample (*N* = 3,292). The data were standardized regression coefficients; ***P < 0.001, **P < 0.01 for coefficients being different from 0.

After adjusting for age, sex, BMI, smoking, alcohol consumption, regular exercise, marriage, caloric intake, family history of CVD, TG, HDL-C, LDL-C, and drug use for hypertension or dyslipidemia, the mediation effects of follow-up cholesterol on the association between the baseline serum calcium and follow-up value of baPWV is shown in Figure 4. The total effect of serum calcium on the baPWV value measured as a standardized regression coefficient (β_Tot_ = 0.060; *P* < 0.001) is estimated without cholesterol in the model. The overall indirect effect of cholesterol was calculated by β_1_ and β_2_ (β_ind_ = 0.013; *P* < 0.001). The percentage of the total effect mediated by cholesterol is estimated at 21.7%.

### Sensitivity Analysis

**Figure 5:**
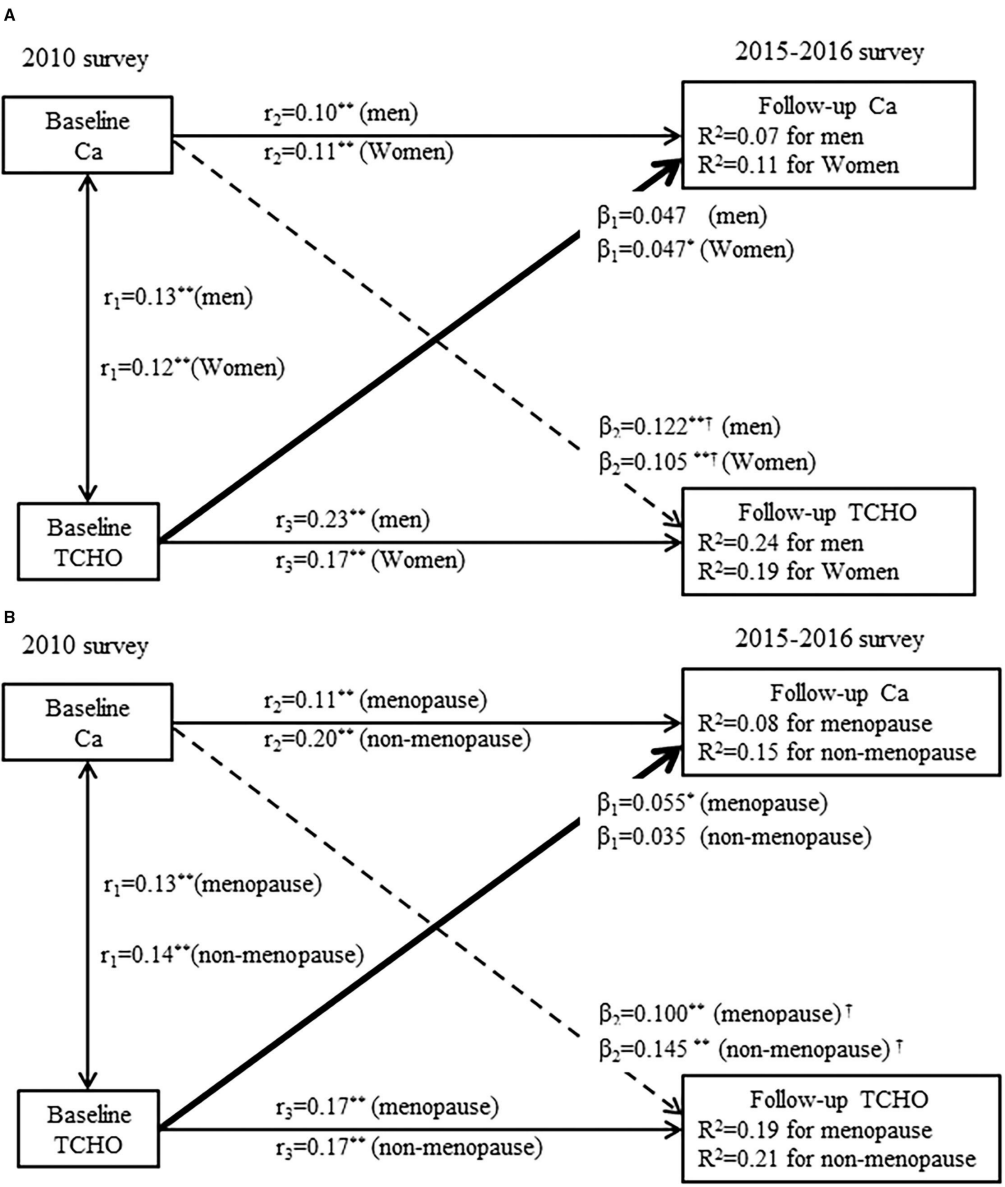
**(A)** A cross-lagged path analysis of serum calcium and total cholesterol in men (*N* = 1202) and women (*N* = 2090); **(B)** A cross-lagged path analysis of serum calcium and total cholesterol in menopause (*N* = 1053) and non-menopausal (*N* = 1037). Adjusted for covariates (covariates included age, BMI, smoking, alcohol consumption, regular exercise, marriage, caloric intake, family history of CVD, TG, HDL-C, LDL-C, and drug use for hypertension or dyslipidemia); β_1_ and β_2_ are the cross-lagged path coefficients, *r*_1_ represents synchronous correlations, *r*_2_ and *r*_3_ represent the tracking correlations, *R*^2^ variance explained. ***P < 0.001 for the coefficients being different from 0.

To further verify whether sex and menopausal status can influence our results,a sensitivity analysis conducts the cross-lagged path analysis in the separate models by sex and menopausal status, with adjustment for covariates (Figures 5A,B). The temporal relationship between the serum calcium and cholesterol do not differ significantly between the men and women or pre-menopause and post-menopause. Another sensitivity analysis calculates the mediation effects of follow-up cholesterol on the association between the baseline calcium and follow-up risk of self-report CVD (Supplementary Figure 1). The results are similar to the mediation effects of follow-up cholesterol on the association between the baseline calcium and follow-up risk CVD diagnosed with the baPWV. The total effect of serum calcium on the CVD is 0.027 (*P* < 0.001). The overall indirect effect for cholesterol is 0.008 (*P* < 0.001). The percentage of the total effect mediated by cholesterol is estimated at 29.6%. The third sensitivity shows that after excluding the participants who took antihypertensive or cholesterol-lowering drugs, the unidirectional relationship from the increased in serum calcium to cholesterol is still observed (Supplementary Figure 2).

## Discussion

This study found that the temporal relationship between the serum calcium and cholesterol was unidirectional, which changes in serum calcium preceded the changes in serum cholesterol. Further, this study found that this unidirectional relationship contributed to the increased in the arterial elasticity or the development of CVD, and the serum cholesterol partially mediated the association between the higher serum calcium levels and greater arterial elasticity or greater risk of the occurrence of CVD.

Inadequate calcium intake is a common health problem in China, calcium supplements are commonly recommended to Chinese, particularly elderly people, to maintain their bone health (19, 20). However, some research have reported an association between the calcium supplementation and increased risk for CVD (21, 22). The serum calcium levels are affected by the calcium supplements (23, 24), recent experimental and epidemiological studies reported that the serum calcium levels could be associated with the pathogenesis of CVD (25). But the mechanism by which the elevated serum calcium levels increase the risk of CVD remains unclear. Although some studies have found a strong inter-correlation between the serum calcium and cholesterol, the temporal relationship between them is not entirely clear. The present study was designed to explore the temporal relationship between the serum calcium and cholesterol. Our analysis confirms the unidirectional relationship between the serum calcium and cholesterol, suggesting that increased in serum calcium preceded increased in serum cholesterol. Some previous basic and animal studies may provide possible mechanisms of this temporal relationship, such as: (1) dietary calcium has a site specific effect on the solubility of bile acids (26); (2) calcium channel blockers reduce cholesterol ester accumulation by increasing intracellular cyclic adenosine monophosphate (27); (3) calcium supplementation significantly inhibited CYP7A1, the key enzyme for cholesterol catabolism, in both mRNA and protein level in estrogen deficiency (6, 19).

Moreover, according to the follow-up status of CVD, we used the ROC curve analysis to determine the cut-off point of the baPWV and performed the cross-lagged path analysis in the people with high and low baPWV to examine whether the unidirectional relationship from serum calcium to cholesterol was associated with the increased arterial elasticity. We found that the path coefficient from serum calcium to cholesterol was significantly greater among the people with the high levels of baPWV than people with low baPWV, suggesting that the higher levels of serum calcium increased cholesterol, resulting in increased arterial elasticity. The common mechanisms for arterial elasticity shared by the high levels of calcium and cholesterol may support the above observations, such as: (1) directly affect the function of vascular endothelial cells; (2) promoting the proliferation of the smooth muscle cells (28–31). *In vitro* and *in vivo* experiments demonstrate that cholesterol enrichment of the smooth muscle cells membrane occurs rapidly and is associated with an increase in calcium permeability, membrane bilayer width, and cell proliferation (32). Other studies have found that in the endothelial cells increasing Ca^2+^ may have detrimental effects by exacerbating TNF-alpha-induced VCAM-1 and monocytes adhesion (33).

Further, the results of mediation analysis also indicated that cholesterol partially mediated the correlation between the serum calcium and further increased the arterial elasticity or incidence of CVD, providing some evidence for the pathogenesis of CVD. These results are consistent with a previous study, which suggested that the accumulation of cholesterol in the smooth muscle cells is an important contributor to the early stimulus initiating atherogenesis in the vessel wall, calcium channel blockers would have protective effect on atherosclerosis (32). Based on the results of mediation analysis, cholesterol may be a possible link between serum calcium and CVD. In accordance with the present results, previous studies have demonstrated that the association between the serum calcium and incident myocardial infarction became non-significant after adjusting for cholesterol (34). Meanwhile, serum calcium appears to be directly related with the lipids and therefore increases individual cardiovascular risk (35). A recent meta-analysis also found that high serum calcium is associated with increased CVD risk, and these associations were attenuated after adjusted for the circulating lipids levels, implying that the circulating lipids could be intermediaries in a chain of calcium and CVD (2, 23).

In this study, we established the temporal relationship between the serum calcium and cholesterol by the cross-lagged path analysis, and further performed the mediation analysis, suggesting that the unidirectional relationship from calcium to cholesterol was linked to incident CVD. However, we also recognized that this study has several limitations. First, this study only included the Asian subjects, which is likely to limit the generalizability of our findings to other ethnic populations. Second, finding from the other studies prove that the genetic variants related to the serum calcium levels is associated with coronary artery disease and myocardial infarction (36). The future studies that include genetic variants are warranted to clarify whether the genetic factors would influence the temporal relationship between the serum calcium and cholesterol. Moreover, this study is observational in natural, we therefore cannot exclude the possibility that the unmeasured covariates would influence the results, especially for the sodium/potassium levels that were not measured in this study (37). Finally, we only measured the baPWV at the follow-up survey. Although we included the participants without the history of CVD, we could not exclude the possibility that the baseline information might influence the mediation effect of elevated serum cholesterol levels on the association between the serum calcium and baPWV. The future studies with measurement of baPWV at multiple time points should be performed to validate these findings.

## Conclusions

In summary, this longitudinal cohort study confirms the unidirectional relationship between the serum calcium and cholesterol, and that the higher serum cholesterol levels partially mediated the association between increased in serum calcium and increased in arterial elasticity or incidence of CVD. These findings add evidence for understanding the potential pathophysiological mechanisms for the serum calcium-CVD association. Also, this study highlights serum calcium as a possible therapeutic target for the prevention of hypercholesterolemia and CVD. Management of the serum calcium levels may be an effective strategy for reducing the risk for CVD, and cholesterol is likely a reasonable therapeutic target of CVD induced by the high serum calcium levels.

## Data Availability Statement

The original contributions presented in the study are included in the article/Supplementary Material, further inquiries can be directed to the corresponding author.

## Ethics Statement

The studies involving human participants were reviewed and approved by the Institutional Review Boards of Harbin Medical University. The patients/participants provided their written informed consent to participate in this study.

## Author Contributions

HS, WW, and YY conceived and designed the experiments. XM and TH performed the experiments. WJ and FD analyzed the data. XM wrote the paper. HS was involved in designing the experiments. All authors have read and approved the final manuscript.

## Funding

This work was supported by the funds from the Harbin Medical University Innovation Research Fund (2020-KYYWF-1443).

## Conflict of Interest

The authors declare that the research was conducted in the absence of any commercial or financial relationships that could be construed as a potential conflict of interest.

## Publisher's Note

All claims expressed in this article are solely those of the authors and do not necessarily represent those of their affiliated organizations, or those of the publisher, the editors and the reviewers. Any product that may be evaluated in this article, or claim that may be made by its manufacturer, is not guaranteed or endorsed by the publisher.
