# Supplementary material for: Temporal Relationship Between Changes in Serum Calcium and Hypercholesteremia and Its Impact on Future Brachial-Ankle Pulse Wave Velocity Levels
Source: Front Nutr. 2021 Nov 17;8:754358. doi: 10.3389/fnut.2021.754358 (PMC8635801; doi:10.3389/fnut.2021.754358)
Supplement: Supplementary file 1 [file Data_Sheet_1.DOCX]

**Supplemental Material**

Indirect effect

*β*_ind_=*β*_1 ×_ *β*_2_=0.008^***^

**Follow-up**

**TCHO**

***β*_2_**

***β*_1_**

Total effect *β*_Tot_=0.027^**^

**Baseline**

**Serum calcium**

Mediation effect by TCHO=29.6%

**Follow-up**

**CVD**

Direct effect

*β*_dir_=0.019^*^

Supplement Figure 1 Mediation effect of follow-up cholesterol on the baseline serum calcium with future risk of cardiovascular disease association with adjustment for covariates (covariates included age, sex, BMI, smoking, alcohol consumption, regular exercise, marriage, caloric intake, family history of cardiovascular disease, TG, HDL-C, LDL-C and drug use for hypertension or dyslipidemia) in the total sample (N =3292). Data were standardized regression coefﬁcients; ^***^*P* < 0.001, ^**^*P*<0.01, ^*^*P* < 0.05 for coefﬁcients being different from 0.


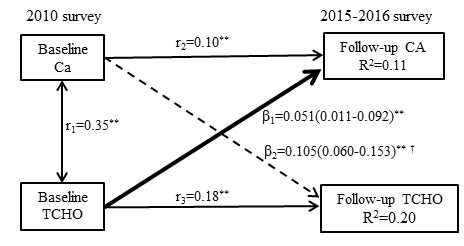


Supplement Figure 2 Cross-lagged path analysis of serum calcium and total cholesterol in low baPWV group and high baPWV group, adjusted for covariates (covariates included age, sex, BMI, smoking, alcohol consumption, regular exercise, marriage, caloric intake, family history of cardiovascular disease, TG, HDL-C, LDL-C) among the participants without taking drugs for anti-hypertension or dyslipidemia (N =2978); *β*_1_ and *β*_2_ are cross-lagged path coefﬁcients, r_1_ represents synchronous correlations, r_2_ and r_3_ represent tracking correlations, R^2^ variance explained. ^**^*P* < 0.001 for coefﬁcients being different from 0. †Difference between *β*_1_ and *β*_2_ for being different from 0.
